# Prognostic Significance of mTOR Expression in Recurrence Following Hepatic Metastasectomy in Colorectal Cancer

**DOI:** 10.3390/life15060877

**Published:** 2025-05-29

**Authors:** Fuat Aksoy, Secil Ak-Aksoy, Ahmet Karamustafaoglu, Cagla Tekin, Melis Ercelik, Berrin Tunca, Busra Oncel Duman, Ozgen Isik, Nesrin Ugras, Ekrem Kaya

**Affiliations:** 1 Department of General Surgery, Faculty of Medicine, Bursa Uludag University, Bursa 16059, Turkey; fuataksoy@uludag.edu.tr (F.A.); drakmo1987@gmail.com (A.K.); or ozgenisik@uludag.edu.tr (O.I.); ekremkaya@uludag.edu.tr (E.K.); 2 Department of Medical Microbiology, Faculty of Medicine, Bursa Uludag University, Bursa 16059, Turkey; 3 Department of Medical Biology, Faculty of Medicine, Bursa Uludag University, Bursa 16059, Turkey; tekincagla9@gmail.com (C.T.); melismutlu95@gmail.com (M.E.); btunca@uludag.edu.tr (B.T.); 4 European Vocational School, Medical Laboratory Techniques Program, Kocaeli Health and Technology University, Kocaeli 41275, Turkey; busra.duman@kocaelisaglik.edu.tr; 5 Department of General Surgery, Faculty of Medicine, Acibadem Mehmet Ali Aydinlar University, Istanbul 34752, Turkey; 6 Department of Pathology, Faculty of Medicine, Bursa Uludag University, Bursa 16059, Turkey; nesrin.ugras@gmail.com

**Keywords:** colorectal cancer, liver metastasis, recurrence, metastasectomy, mTOR

## Abstract

Surgery is one of the most effective treatment methods for liver metastases developing from primary colorectal cancer (CRC). Despite the widespread application of surgical approaches, recurrence rates remain substantial. Although chemotherapy is frequently employed, the supporting evidence for its efficacy in this context remains inconclusive. In the present study, we aimed to identify potential predictors of post-metastasectomy recurrence by analyzing clinical, pathological, and molecular features of both primary colorectal tumors and their corresponding hepatic metastases. Specifically, we evaluated the expression of epithelial–mesenchymal transition (EMT) markers, cancer stem cell (CSC) markers, and selected oncogenic mRNAs (RAS, mTOR, and CMYC) in tissue samples from 84 patients. RAS and CMYC are well-known proto-oncogenes involved in cell proliferation and survival, while mTOR functions as a central regulator of cell growth and metabolism. Following liver metastasectomy, intra-hepatic recurrence was observed in 40.5% of the cases. Among the molecular markers analyzed, the EMT transcription factor SNAIL—which plays a critical role in cancer cell invasion and metastasis—and mTOR exhibited significantly elevated expression in metastatic lesions from patients who experienced recurrence. While SNAIL expression did not show a clear association with the time to recurrence, increased mTOR expression in metastatic liver tissue was significantly associated with both shorter recurrence-free survival and diminished overall survival (*p* < 0.001). Results showed that *mTOR* expression levels could be a clinically relevant predictive indicator of remnant liver recurrence. In patients with liver metastases, the use of *mTOR* inhibitors may be considered after hepatic metastasectomy.

## 1. Introduction

Colorectal cancer (CRC) stands out as the predominant malignant tumor affecting the digestive tract, contributing significantly to global cancer-related mortality [1]. CRC often manifests metastatic disease, most frequently in the liver, affecting at least 40% of patients who develop colorectal liver metastases over the course of their illness [2]. Conversion of initially unresectable liver metastases to resectable status can significantly improve outcomes, with reported 5-year survival rates rising from approximately 30% to as high as 65%. In contrast, the 5-year survival rate for patients who do not receive any surgical or medical intervention remains below 2% [3,4,5]. Therefore, when technically and clinically feasible, surgical removal of isolated liver metastases remains the preferred therapeutic approach. Nevertheless, recurrence rates after this procedure are high, making systemic treatment ideal to achieve longer survival [4]. While perioperative and adjuvant therapies have demonstrated some effectiveness in patients with resectable hepatic metastases, the role of systemic therapy following curative liver resection remains ambiguous [5]. This uncertainty stems from the limited number of robust clinical trials and early termination of some studies due to inadequate patient enrollment, resulting in inconsistent findings across the literature. As such, there is no conclusive evidence confirming that systemic treatment offers a significant survival advantage over observation alone. Nevertheless, a recent meta-analysis incorporating both randomized controlled trials and observational studies reported improved overall survival (OS) and recurrence-free survival in patients who received systemic chemotherapy following liver metastasectomy. Despite these findings, the optimal timing—whether neoadjuvant, perioperative, or adjuvant—and the most effective chemotherapy regimen are still subjects of ongoing discussion. Moreover, a reliable prognostic biomarker to guide treatment decisions hepatic (liver) metastasectomy is currently lacking, leaving the most appropriate therapeutic strategy for these patients a matter of debate [6,7].

The aim of the study was to examine molecular markers that can be used to determine the development of recurrence after metastasectomy. In this study, both primary and metastatic tumor tissues of the same patient were examined and an answer was sought to the question of whether the primary tumor or the metastatic tumor should be used to predict prognosis.

## 2. Materials and Methods

### Patient Selection and Sample Collection

Between December 2011 and April 2018, patients who underwent surgery for primary CRC and Liver Metastases at the University Hospital (Bursa Uludag University) were enrolled in the study. Clinical and follow-up data were retrospectively retrieved from the archives of Uludağ University Faculty of Medicine and the Department of General Surgery. Key demographic and tumor-related variables—including patient age, sex, tumor localization, and pathological staging based on the TNM classification system—were systematically reviewed. The study population was limited to patients diagnosed with sporadic colorectal cancer (CRC). Patients were eligible if they either developed recurrence or remained disease-free for a minimum of five years after surgical intervention. Based on these clinical outcomes, participants were categorized into progressive or stable disease groups. Time to recurrence and disease-free interval were defined, respectively, as the period from surgery to either confirmed tumor relapse or last known follow-up without recurrence. Disease-free survival (DFS) referred to survival without radiological or clinical evidence of recurrence at the latest documented visit. Additionally, median overall survival (OS) and median progression-free survival (PFS) were calculated. The study received ethical approval from the institutional review board and was conducted in accordance with the principles outlined in the Declaration of Helsinki. Inclusion criteria required the availability of both primary tumor and matched metastatic liver tissue samples. Patients were excluded if they had achieved a complete pathological response after neoadjuvant chemotherapy, had synchronous malignancies, or had undergone resection with positive surgical margins. In total, 84 patients meeting these criteria were enrolled in the study. All colorectal cancer surgeries were performed according to standard oncologic principles. Depending on tumor location, patients underwent low anterior resection, right or left hemicolectomy, or sigmoidectomy with regional lymphadenectomy. Resections were performed with the intent of achieving R0 margins, and no patients included in this study had macroscopic residual disease after colorectal surgery. Liver metastasectomies were carried out by experienced hepatobiliary surgeons following preoperative radiological assessment and multidisciplinary tumor board evaluation. The choice of anatomical (e.g., segmentectomy, lobectomy) or non-anatomical (wedge resection) hepatectomy was based on the size, location, and number of metastases, as well as future liver remnant volume. In select cases, Pringle’s maneuver (intermittent portal triad clamping) was employed to minimize intraoperative bleeding. All procedures were performed with curative intent, and patients with R2 resections or positive surgical margins were excluded from the study. Surgical specimens, including primary tumor tissues and matched metastases, were collected by December 2022. The working groups were as follows; normal colon tissue (n = 8), CRC with liver metastases (n = 84), normal liver tissue (n = 12), liver metastasis tissues of CRC tumors. Formalin-fixed and paraffin-embedded (FFPE) specimens were retrieved from the Pathology Department of the University Hospital. FFPE tumor and normal samples were deparaffinized using xylene and ethanol. Extraction of total RNA was performed immediately after deparaffinization using the Recover All™ Total Nucleic Acid Isolation Kit for FFPE (Ambion™, Austin, TX, USA) according to the manufacturer’s protocol and immediately stored at −80 °C until the next step. Nucleic acid concentration, A260/280, and A260/230 ratios were measured using NanoDrop One/OneC Spectrophotometer (Thermo Fisher Scientific, Wilmington, DE, USA). For downstream applications, approximately 300 ng of total RNA was used to synthesize complementary DNA (cDNA) using the High-Capacity cDNA Reverse Transcription Kit (Applied Biosystems, Foster City, CA, USA). Gene-specific primers, including those for the housekeeping gene GAPDH, were carefully selected and optimized for each target, as detailed in Table 1. Only samples with quantification cycle (Ct) values below 40 for all target transcripts were considered acceptable for further analysis based on amplification curve assessment. Gene expression levels were quantified using the 2^−ΔΔCt method, where ΔCt represents the difference between the Ct value of the target gene and that of the reference (housekeeping) gene. The resulting fold-change reflects relative gene expression in comparison to a control sample. For transcripts showing statistically significant differential expression, receiver operating characteristic (ROC) curve analysis was performed using GraphPad Prism to determine optimal cut-off values, sensitivity, and specificity. Patients were dichotomized into two groups based on whether their 2^−ΔΔCt values were above or below the established cut-off thresholds. Associations between gene expression profiles and clinicopathological variables were examined using the chi-square test. Kaplan–Meier survival curves were constructed to evaluate disease-free survival (DFS), with differences assessed by the Log-rank test. All statistical analyses were conducted using GraphPad Prism version 9.5.1 (GraphPad Software, San Diego, CA, USA) was used for statistical analysis and IBM SPSS Statistics version 23.0. Parametric or non-parametric tests were selected based on the distribution characteristics of the data. Relationships between continuous variables were evaluated using Pearson or Spearman correlation coefficients, as appropriate. Continuous variables were presented as median and interquartile range (IQR), as recommended for non-normally distributed data. A *p*-value less than 0.05 was considered indicative of statistical significance throughout the analyses (Supplementary Materials).

## 3. Results

### 3.1. Recurrence Patterns and Clinical Correlates Following Liver Metastasectomy

This study included 84 adult individuals diagnosed with colorectal cancer (CRC) and liver metastases who underwent surgical removal of metastatic lesions. Among them, 17 patients (20.2%) had metastasectomy performed concurrently with the initial colorectal tumor resection and were categorized as having synchronous metastases. The remaining 67 patients (79.8%) underwent metastasectomy at a later stage, following disease recurrence, and were classified as metachronous cases. The majority of patients were men (71.4%; 60/84), with a median age of 58 years at the time of CRC diagnosis. After synchronous metastasectomy, locoregional recurrence developed in 10 patients. Twenty-four patients in the metachronous group had locoregional recurrence after metastasectomy. As described in the Methods section, patients with primary CRC were divided into two groups depending on recurrence after liver metastasectomy. A comparison of the patient demographics, tumor characteristics, and utilization of neoadjuvant and adjuvant therapies of the recurrent and non-recurrent groups is summarized in Table 1.

The median DFS was 91.0 months for the non-recurrent group and 15.5 months for the recurrent group. The recurrence development of the tumors differed significantly by gender (*p* = 0.017). Significantly more men had recurrent tumors (n = 25; 85.3%) than did women (n = 5; 14.7%). According to the chi-square analysis, the rate of recurrence was higher in those who did not receive adjuvant treatment (n = 40, 80.0%; *p* = 0.032).There were no significant differences in other clinicopathological factors between the two groups (Table 1).

It is reported that rectal cancer is different from colon cancer in etiology, genetics, anatomy, clinical manifestation, biological feature, treatment response and clinical outcomes. Therefore, we examined the distribution of chemotherapy receipt, recurrence status according to tumor location (Table 2).

### 3.2. Molecular Features of Primary Tumors and Metastatic Tumors

**Figure 1:**
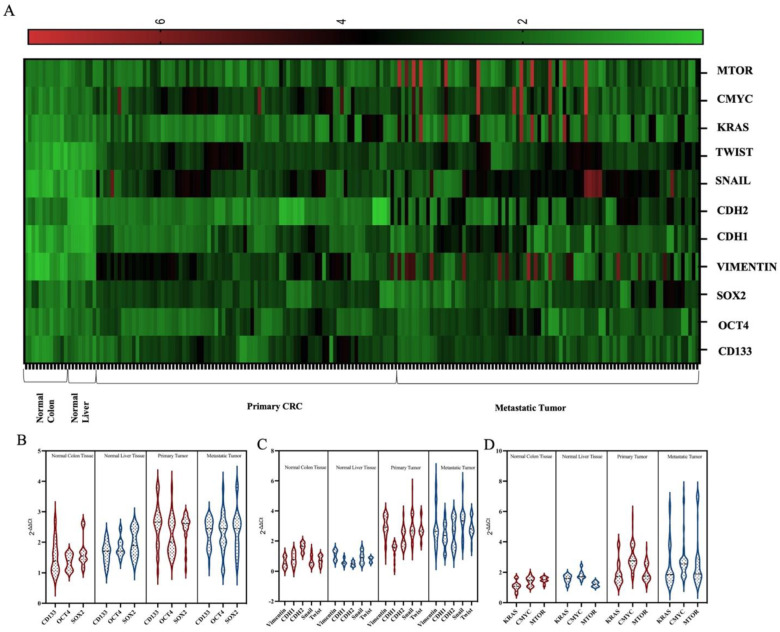
Differential gene expression in all samples. Heatmap showing differential gene expression between yellow and red. Red and green shadings represent higher and lower relative expression levels, respectively (**A**). The expression profiles of CSC markers in all groups (**B**). The expression profiles of EMT markers in all tissues (**C**). The expression profiles of *KRAS*, *CMYC* and *mTOR* in all tissues (**D**).

CSC markers, EMT mechanism and *mTOR*, *RAS*, *MYC* mRNAs were examined to determine the molecular characteristics of tumors that recurrence after metastasectomy. Molecular analyses were first performed on primary tumor tissue (CRC) by comparing it with normal colon tissue. Afterwards, analyzes were performed in metastatic liver tumors by comparing them with normal liver tissue (Figure 1A).

First, it was determined that *SOX2*, *OCT4* and *CD133* expressions were higher in CRC tumors compared to normal colon tissue (*p* < 0.05, Figure 1). When HCC was compared with normal liver tissue, it was determined that the expression of three CSC markers was higher in tumor tissue (*p* < 0.05, Figure 1B).

The EMT mechanism was examined by analyzing the expression levels of EMT markers *VIMENTIN*, *CDH1*, *CDH2*, *SNAIL* and *TWIST* mRNAs. When 84 CRC tissue samples with liver metastasis were compared with 12 normal colon tissue samples, it was determined that the EMT process was active (*p* < 0.05). *VIMENTIN*, *SNAIL* and *TWIST* were found to be highly expressed when liver metastasis tissues were compared with normal liver tissue (*p* < 0.001, Figure 1C).

The expression profiles of *mTOR*, *MYC* and *RAS*, which are important signaling pathways in cancer progression, were examined at the mRNA level. When primary CRC tissues were compared with normal colon tissues, it was determined that the *KRAS* and *MYC* were highly expressed (*p* < 0.05). In the metastatic tumor, *mTOR*, *KRAS* and *MYC* were found to be up regulated compared with normal liver tissues (*p* < 0.05, Figure 1D).

**Figure 2:**
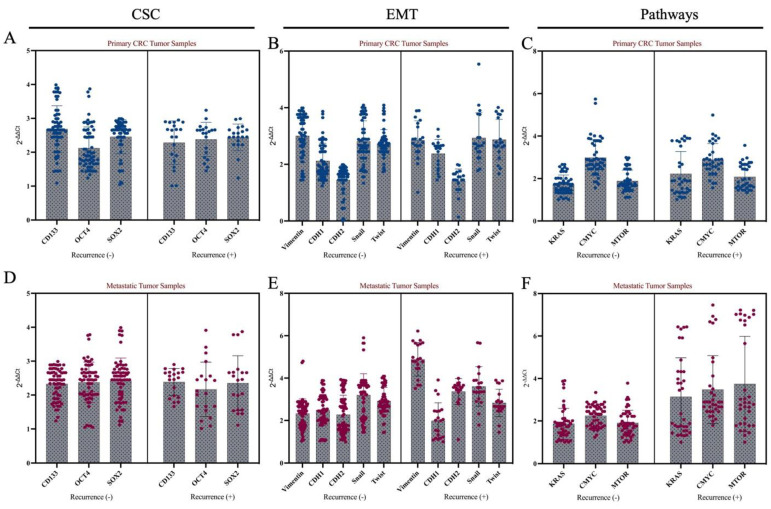
Expression profiles of CSC, EMT, and oncogenic pathway-related genes in primary and metastatic tumor tissues of colorectal cancer patients according to recurrence status. Panels (**A**–**C**) represent gene expression in primary CRC tumor tissues, while Panels (**D**–**F**) show expression levels in matched metastatic liver tumor tissues.

Afterwards, tumors were divided into two groups according to their recurrence status after metastasectomy, and CSC and EMT markers, and *mTOR*, *RAS*, *MYC* were examined. No statistically significant change in expression of CSC markers was observed between the two groups (*p* > 0.05, Figure 2A). In addition, when tumor tissues were grouped according to recurrence status, no difference was found between the two groups in any of the EMT markers in the primary tumor tissue (*p* > 0.05, Figure 2B). There was no difference in *KRAS*, *MYC* or *mTOR* expression in primary colon tumors that developed after metastasectomy compared to tumors that did not recur (*p* > 0.05, Figure 2C).

In metastatic liver tissues, high expression of *SNAIL* was found to be significant in recurrent liver tumors after metastasectomy (*p* < 0.001). Moreover, *mTOR* expression was found to be high in those who developed recurrence after metastasectomy (Figure 2).

### 3.3. Pathological and Clinical Characteristics of Patients with High SNAIL and mTOR Expression

**Figure 3:**
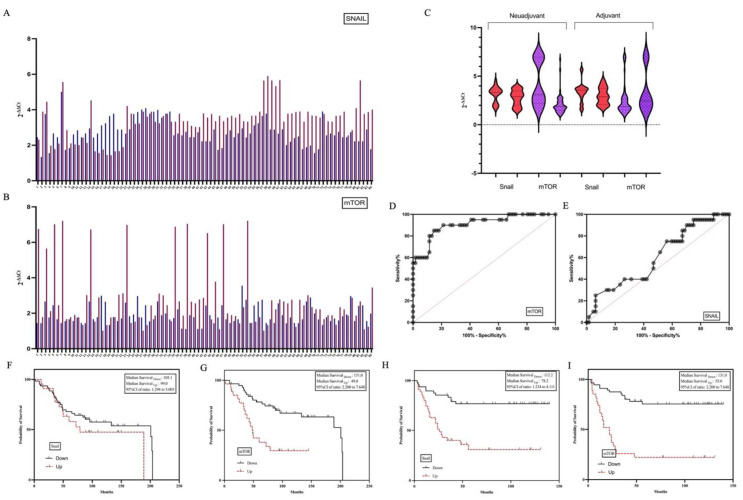
Matched primary tumors and distal metastases from 85 CRC patients were collected and deregulated genes were analyzed by comparing distal metastases with matched primary tumors. Expressions of *SNAIL* (**A**) and *mTOR* (**B**) were found to be higher in metastatic HCC tissues than in the primary CRC. It was determined that mTOR expression decreased in the metastatectomy material of the patient group receiving neoadjuvant but not significant (**C**). The cutoff value of *mTOR* expression in metastatic tissues was determined as 2.745, and *SNAIL* was determined as 2.612 (**D**,**E**). The effect of *SNAIL* expression profiles on the relapse time was not determined (**F**,**H**). High expression in metastatic liver tissue was associated with a short recurrence time and short overall survival (**G**,**I**).

Then, the expression profiles of *SNAIL* and *mTOR* were examined in the primary and their matched metastatic tumors of the patients and both mRNAs were determined to be higher in liver tissues (Figure 3A,B). The administered chemotherapy protocols included capecitabine combined with oxaliplatin (XELOX) in 12 patients, a regimen of fluorouracil, leucovorin, and irinotecan (FOLFIRI) in 20 patients, and fluorouracil, leucovorin, and oxaliplatin (FOLFOX-6) in 11 patients. Among the study cohort, 17 individuals received neoadjuvant chemotherapy, while 26 were treated in the adjuvant setting. Importantly, all patients underwent treatment with chemotherapy regimens based on 5-fluorouracil (5-FU). There was no difference in recurrence after metastasectomy between patients who received and did not receive a neoadjuvant. When the effects of adjuvant and neoadjuvant chemotherapy on gene expressions were examined, no effect of preoperative/postoperative chemotherapy was determined in the CRC tissues. However, it was determined that *mTOR* expression decreased in the metastatectomy material of the patient group receiving neoadjuvant, statistical significance was not determined (Figure 3C).

Cut off values of *SNAIL* and *mTOR* expressions in the metastatic tumor were determined and the patients were divided into two groups: high expression and low expression. Patients had a median follow-up of 60.11 months (interquartile range: 1 to 210 months). Forty patients died. The average lifespan was 84.75 months. A relationship was determined between adjuvant treatment and a long survival time. Recurrence was detected in 34 patients. The mean recurrence time was determined as 46.506 (minimum, maximum; 6–57, Std. Eror: 5074). The effect of *SNAIL* expression profiles on the relapse time was not determined (Figure 3F,H). However, high *mTOR* expression (cut off: 2.745) in metastatic liver tissue was associated with a short recurrence time and short overall survival (*p* < 0.001, Figure 3G,I).

## 4. Discussion

The survival benefit gained from metastasectomy in patients with liver metastases from CRC is well established [8]. Nevertheless, the recurrence of tumors following this procedure is currently an important problem [9,10,11]. In our study, we aimed to identify active molecular markers in the development of recurrence after metastasectomy. In this context, we examined both primary and metastatic tumor tissues of 84 CRC patients with liver metastasis. To predict recurrence after metastasis and to determine an appropriate therapeutic option, it is important to first understand the molecular mechanism of metastasis.

EMTs and CSCs are defined as the most important molecular mechanisms involved in cancer metastasis. There is substantial evidence indicating that the metastasis of cancer cells begins from cells with the most characteristics of stem cells, called CSCs [11]. CSCs are known to be cells that show self-renewal ability and asymmetric division. These cells are also associated with cancer cell growths and metastasis and tumor recurrence following treatment [12]. Studies have recently shown that targeting CSC can be an effective treatment strategy for cancer treatment [13,14]. When we examined EMT and CSC markers, they were all found to be highly expressed in both primary and metastatic tissues compared to normal tissues in our study. OCT4, a pivotal regulator involved in epithelial–mesenchymal transition (EMT), has been associated with the pathogenesis of multiple malignancies, such as gastric, breast, and non-small cell lung cancers, as well as gliomas and esophageal squamous cell carcinoma [15]. In a study by Saigusa et al., elevated OCT4 expression was linked to an increased risk of distant recurrence in rectal cancer patients following chemoradiotherapy [16]. Similarly, Gazouli et al. reported that OCT4 expression was significantly upregulated in colorectal cancer (CRC) tissues compared to adjacent normal colon tissues, with expression levels positively correlating with tumor stage [17]. However, despite these findings, the functional significance of OCT4 in liver metastases originating from CRC and its underlying molecular mechanisms remain to be fully elucidated. In the present study, we found that OCT4 expression was upregulated in metastatic tumor compared with normal liver tissue. However, high OCT4 expression was not associated with poor prognosis after metastasectomy. In the current study, other CSC markers, SOX2 and CD133, were found to be highly expressed in CRC and metastatic liver tumors, but no significant expression change was detected in these markers in recurrence after metastasectomy. The results obtained show that the CSC process plays a role in liver metastasis in CRC, but they may not be effective in recurrence of the disease after metastasectomy.

Recent studies have shown that EMT can have a key role in cancer cell metastasis [18,19,20]. During EMT, cells lose cell–cell and cell–extracellular matrix adhesion, acquiring mesenchymal cell phenotypes. This allows the detachment of cells from the primary tumor and the separation of the surrounding tissues and distant organs [20,21]. In the current study, we analyzed the EMT process by examining the expressions of *VIMENTIN*, *CDH1*, *CDH2*, *SNAIL* and *TWIST*. While no change in *CDH1* and *CDH2* expression was detected in the primary tumor tissue compared to normal colon tissue, vimentin *SNAIL* and *TWIS* showed high expression in the tumor tissue. In metastatic tumor tissues, it was determined that all EMT markers were expressed higher compared to normal liver tissue.

Liver metastases from CRC tumors are one of the most studied topics in this field [1,2]. However, most metastasis hypotheses are mainly based on observations of cell culture, experiments of transplant animal models, primary cancer tissues [22,23]. They seldom focus on metastatic tissues because metastatic tissues are difficult to be obtained by biopsy or resection. Thus, they have some limitations in terms of understanding the metastatic process. To gain a comprehensive view of metastases, priority should be given to paired analysis of primary tumors and metastases. Now this thought has been recognized and some institutions have conducted studies on comparisons between primary tumors and metastases at multiple levels by traditional and modern technologies. In our study, we examined molecular markers in primary CRC and their metastatic HCC tissues and we determined that SNAIL and mTOR increased in metastatic tumors compared to matching CRC tissue.

mTOR, the mammalian target of rapamycin, has emerged as a potential target for drug development, particularly due to the fact that it plays such a crucial role in cancer biology [24]. The mTOR pathway is upregulated in HCC tissue samples compared to the surrounding cirrhotic liver tissue [24]. In addition, the activation of mTOR is more intense in the tumor edge, thus reinforcing its role in HCC proliferation and spreading [25]. However, there are no studies of mTOR with primary CRC and metastatic HCC tumors. In our study, we determined that mTOR expression increased even more in metastatic tumors.

Tumors were divided into two groups according to their recurrence status after metastasectomy. In our study, we determined that high *SNAIL* and *mTOR* expressions were significant among all parameters evaluated in the development of recurrence after metastasectomy. In our stduy, the 5-year DFS rates were 42.3%, OS rates were 70.2%. When the effects of Snail and mTOR on DFS and OS are examined, it is seen that high expression of mTOR is associated with short DFS. 

mTOR expression can accurately predict remnant liver recurrence after metastasectomy.

Although there are a lot of studies in the literature comparing primary tumors and metastatic tumors, there are no studies within this scope which regard the development of recurrence after metastasectomy. Comparisons of metastases matched with primary cancer are of interest to researchers, but have not produced significant findings. Previous research has demonstrated a high degree of genetic similarity between primary tumors and their corresponding metastases, supporting the notion that metastatic lesions often originate from a common clonal source and retain many of the core molecular characteristics of the primary tumor. This insight is particularly valuable for determining the origin of metastatic tumors and guiding therapeutic strategies based on the biology of the primary lesion. However, emerging evidence also indicates that metastases may acquire new features influenced by the distinct microenvironment of the secondary site, necessitating adaptive changes that promote tumor cell survival and colonization in these new contexts.

This retrospective study had several limitations. That is, it was performed at a single center. Colon and rectum tumors were evaluated together. Both metachronous and synchronous metastases were included in the study. While ROC curve analyses were employed to evaluate the predictive performance of SNAIL and mTOR expression in relation to recurrence after liver metastasectomy, we acknowledge that the relatively small sample size limits the robustness and generalizability of these findings. The AUC values and derived cut-off points should be interpreted cautiously and primarily serve an exploratory purpose. Future studies with larger patient cohorts are warranted to validate the predictive accuracy of these biomarkers and to establish clinically applicable thresholds.

## 5. Conclusions

In studies conducted over the last 10 years, colon and rectum tumors are now considered separate cancers. For this reason, the chemotherapy regimens given also differ between the two cancers. However, in liver metastasis studies, colon and rectum are evaluated together as colorectal tumors. In our study, when we evaluated the tumors separately as colon and rectum, we determined that there was no statistically significant difference between the two tumor groups in the development of recurrence after metastasectomy. However, SNAIL and mTOR expression profiles did not differ between colon and rectum tumors. This result shows that tumor localization has no effect on recurrent recurrences of liver metastases originating from primary colorectal cancer. Furthermore, it may Be thought that tumors’ biologic behavior in the metastatic era can be changed.

The management of CRC liver metastasis poses several challenges for surgeons due to the complexity of the disease. In some cases, advanced liver metastases may be deemed unresectable. Surgeons face the challenge of determining alternative treatment strategies, such as chemotherapy, targeted therapy, or other liver-directed therapies. Even with successful resection of liver metastases, there is a risk of recurrence. Surgeons must weigh the benefits of surgery against the potential for future metastatic spread. Following established guidelines for the management of CRC liver metastasis is essential. Surgeons need to consider evidence-based practices and multidisciplinary input to optimize patient outcomes. Therefore, a better understanding of tumor biology in liver metastasis in CRC is needed.

Our main aim in this study was to find a parameter that can be used to identify patients with recurrence after metastasectomy. In summary, we first report that the statistically significant upregulation of *SNAIL* and *mTOR* were an independent risk factor for recurrence of CRC patients with liver metastasis following metastasectomy. As a result of our study, we emphasize that different treatment options such as mTOR inhibitors may be considered instead of metastasectomy, especially in patients with determined recurrence potential.

## Figures and Tables

**Table 1:** Patient demographics and tumor characteristics.

Features	Non-Recurrent	Recurrent	*p* Value
	n = 50 (59.5%)	n = 34 (40.5%)	
*Sex*			**0.017**
Male	31 (62.0%)	25 (85.3%)	
Female	19 (38.0%)	5 (14.7%)	
*Age (median, IQR)*	60.0 (55.0–68.5)	59.0 (53.0–65.0)	0.226
*Primary Tumor Site*			0.513
Sigmoid	16 (32.0%)	11 (32.4%)	
Left colon	6 (12.0%)	2 (5.9%)	
Right colon	6 (12.0%)	4 (11.8%)	
Transvers colon	2 (4.0%)	1 (2.9%)	
Rectum	20 (40.0%)	16 (47.1%)	
*Primary Tumor (T)*			0.507
1	1 (2.0%)	1 (2.9%)	
2	4 (8.0%)	6 (17.6%)	
3	39 (78.0%)	22 (67.7%)	
4	6 (12.0%)	5 (14.7%)	
*Regional Lymph Node (N)*			0.711
0	17 (34.0%)	12 (35.3%)	
1	15 (30.0%)	7 (20.6%)	
2	18 (36.0%)	15 (44.1%)	
*Lymphatic Invasion*			0.502
Absence	35 (70.%)	23 (67.6%)	
Presence	15 (30.0%)	11 (32.4%)	
*Perineural Invasion*			0.302
Absence	39 (78.0%)	24 (70.6%)	
Presence	11 (22.0%)	10 (29.4%)	
*Vascular Invasion*			0.375
Absence	40 (80.0%)	29 (85.3%)	
Presence	10 (20.0%)	5 (14.7%)	
*Type of Liver Metastasis*			0.173
Metachronous	43 (86.0%)	24 (70.6%)	
Synchronous	7 (14.0%)	10 (29.4%)	
*Adjuvant Therapy*			*0.032*
Absence	10 (20.0%)	14 (41.2%)	
Presence	40 (80.0%)	20 (58.8%)	
*Neoadjuvant Therapy*			0.363
Absence	41 (82.0%)	26 (76.5%)	
Presence	9 (18.0%)	8 (23.5%)	

*p*-values were calculated using non-parametric tests (Fisher’s Exact Test for categorical variables and Mann–Whitney U test for continuous variables), as appropriate. Values in bold are statistically significant at *p* < 0.05.

**Table 2:** Relationship between Tumor Localization and chemotherapy, recurrence and mRNA expression profiles.

Localization	Neoadjuvant		Adjuvant		Recurrence After Metastasectomy		Snail		mTOR	
	(−)	(+)	(−)	(+)	(−)	(+)	Down	Up	Down	Up
Rectum	25 (69.4)	11 (%30.6)	11 (30.6)	25 (69.4)	20 (55.6)	16 (44.4)	15 (41.7)	21 (58.3)	21 (58.3)	15 (41.7)
Sigmoid	23 (85.2)	4 (14.8)	10 (37)	17 (63)	16 (59.3)	11 (40.7)	9 (33.3)	18 (66.7)	20 (74.1)	7 (25.9)
Left Colon	8 (100)	0 (0)	0 (0)	8 (100)	6 (75)	2 (25)	2 (25)	6 (75)	7 (87.5)	1 (12.5)
Right Colon	9 (90%)	1 (10)	3 (30)	7 (70)	6 (60)	4 (40)	3 (30)	7 (70)	7 (70)	3 (30)
Transvers Colon	2 (66.7)	1 (33.3)	0 (0)	3 (100)	2 (66.7)	1 (33.3)	2 (66.7)	1 (33.3)	2 (66.7)	1 (33.3)

## Data Availability

All data generated or analyzed during this study are included in this published article. The data that support the findings of this study are available from the corresponding author upon request.

## Supplementary Materials

The following supporting information can be downloaded at: https://www.mdpi.com/article/10.3390/life15060877/s1, Figure S1: Flowchart illustrating patient selection and inclusion in the study.

## Abbreviations

CRC	Colorectal cancer
OS	Overall survival
FFPE	Formalin-fixed and paraffin-embedded
CSC	Cancer stem cell
EMT	Epithelial–mesenchymal transition

## References

[1] Martin J., Petrillo A., Smyth E.C., Shaida N., Khwaja S., Cheow H., Duckworth A., Heister P., Praseedom R., Jah A. (2020). Colorectal liver metastases: Current management and future perspectives. World J. Clin. Oncol..

[2] Feng Q.Y., Wei Y., Chen J.W., Chang W.J., Ye L.C., Zhu D.X., Xu J.M. (2014). Anti-EGFR and anti-VEGF agents: Important targeted therapies of colorectal liver metastases. World J. Gastroenterol..

[3] Wang Y., Zhong X., He X., Hu Z., Huang H., Chen J., Chen K., Zhao S., Wei P., Li D. (2023). Liver metastasis from colorectal cancer: Pathogenetic development, immune landscape of the tumour microenvironment and therapeutic approaches. J. Exp. Clin. Cancer Res. CR.

[4] Valderrama-Trevino A.I., Barrera-Mera B., Ceballos-Villalva J.C., Montalvo-Jave E.E. (2017). Hepatic metastasis from colorectal cancer. Eur. J. Hepato-Gastroenterol..

[5] Adam R., Delvart V., Pascal G., Valeanu A., Castaing D., Azoulay D., Giacchetti S., Paule B., Kunstlinger F., Ghémard O. (2004). Rescue surgery for unresectable colorectal liver metastases downstaged by chemotherapy: A model to predict long-term survival. Ann. Surg..

[6] Kanemitsu Y., Mizusawa J., Inaba Y., Hamaguchi T., Shida D., Ohue M., Komori K., Shiomi A., Shiozawa M., Watanabe J. (2021). Hepatectomy Followed by mFOLFOX6 Versus Hepatectomy Alone for Liver-Only Metastatic Colorectal Cancer (JCOG0603): A Phase II or III Randomized Controlled Trial. J. Clin. Oncol..

[7] Kopetz S., Chang G.J., Overman M.J., Eng C., Sargent D.J., Larson D.W., Grothey A., Vauthey J.-N., Nagorney D.M., McWilliams R.R. (2009). Improved Survival in Metastatic Colorectal Cancer Is Associated with Adoption of Hepatic Resection and Improved Chemotherapy. J. Clin. Oncol..

[8] Benson A.B., Venook A.P., Al-Hawary M.M., Arain M.A., Chen Y.J., Ciombor K.K., Cohen S., Cooper H.S., Deming D., Farkas L. (2021). Colon Cancer, Version 2.2021, NCCN Clinical Practice Guidelines in Oncology. J. Natl. Compr. Canc. Netw..

[9] (2020). 2020 Exceptional Surveillance of Colorectal Cancer (NICE Guideline NG151).

[10] Van Cutsem E., Cervantes A., Adam R., Sobrero A., Van Krieken J.H., Aderka D., Aranda Aguilar E., Bardelli A., Benson A., Bodoky G. (2016). ESMO consensus guidelines for the management of patients with metastatic colorectal cancer. Ann. Oncol. Off. J. Eur. Soc. Med. Oncol..

[11] Tang K.D., Holzapfel B.M., Liu J., Lee T.K., Ma S., Jovanovic L., An J., Russell P.J., Clements J.A., Hutmacher D.W. (2016). Tie-2 regulates the stemness and metastatic properties of prostate cancer cells. Oncotarget.

[12] Yoshizumi A., Kuboki S., Takayashiki T., Takano S., Takayanagi R., Sonoda I., Ohtsuka M. (2023). Tspan15-ADAM10 signalling enhances cancer stem cell-like properties and induces chemoresistance via Notch1 activation in ICC. Liver Int..

[13] Wang J., Zhang B., Chen X., Xin Y., Li K., Zhang C., Tang K., Tan Y. (2024). Cell mechanics regulate the migration and invasion of hepatocellular carcinoma cells via JNK signaling. Acta Biomater..

[14] Hu J., Chen K., Hong F., Gao G., Dai X., Yin H. (2024). METTL3 facilitates stemness properties and tumorigenicity of cancer stem cells in hepatocellular carcinoma through the SOCS3/JAK2/STAT3 signaling pathway. Cancer Gene Ther..

[15] Ye P., Chi X., Yan X., Wu F., Liang Z., Yang W.H. (2022). Alanine-Glyoxylate Aminotransferase Sustains Cancer Stemness Properties through the Upregulation of SOX2 and OCT4 in Hepatocellular Carcinoma Cells. Biomolecules.

[16] Saigusa S., Tanaka K., Toiyama Y., Yokoe T., Okugawa Y., Ioue Y., Miki C., Kusunoki M. (2009). Correlation of CD133, OCT4, and SOX2 in rectal cancer and their association with distant recurrence after chemoradiotherapy. Ann. Surg. Oncol..

[17] Gazouli M., Roubelakis M.G., Theodoropoulos G.E., Papailiou J., Vaiopoulou A., Pappa K.I., Nikiteas N., Anagnou N.P. (2011). Anagnou OCT4 spliced variant OCT4B1 is expressed in human colorectal cancer. Mol. Carcinog..

[18] Liu W., Tang J., Gao W., Sun J., Liu G., Zhou J. (2024). PPP2R1B abolishes colorectal cancer liver metastasis and sensitizes Oxaliplatin by inhibiting MAPK/ERK signaling pathway. Cancer Cell Int..

[19] Niu W., Liu Q., Huo X., Luo Y., Zhang X. (2024). TL1A promotes metastasis and EMT process of colorectal cancer. Heliyon.

[20] Aksoy S.A., Tunca B., Erçelik M., Tezcan G., Ozturk E., Cecener G., Ugras N., Yilmazlar T., Yerci O. (2022). Early-stage colon cancer with high MALAT1 expression is associated with the 5-Fluorouracil resistance and future metastasis. Mol. Biol. Rep..

[21] De Craene B., Berx G. (2013). Regulatory networks defining EMT during cancer initiation and progression. Nat. Rev. Cancer.

[22] Hata N., Shigeyasu K., Umeda Y., Yano S., Takeda S., Yoshida K., Fuji T., Yoshida R., Yasui K., Umeda H. (2023). ADAR1 is a promising risk stratification biomarker of remnant liver recurrence after hepatic metastasectomy for colorectal cancer. Sci. Rep..

[23] Wang Y.Y., Xin Z.C., Wang K. (2023). Impact of Molecular Status on Metastasectomy of Colorectal Cancer Liver Metastases. Clin. Colon Rectal Surg..

[24] Sun M., Jiang Z., Gu P., Guo B., Li J., Cheng S., Ba Q., Wang H. (2023). Cadmium promotes colorectal cancer metastasis through EGFR/Akt/mTOR signaling cascade and dynamics. Sci. Total Environ..

[25] Chen G., Tian T.T., Wang F.Q., Pan C.S., Sun K., Wang X.Y., Yang B., Yang Z., Tang D.X., Han J.Y. (2024). Chanling Gao suppresses colorectal cancer via PI3K/Akt/mTOR pathway modulation and enhances quality of survival. Environ. Toxicol..

