# Supplementary figures and images for: Prognostic Significance of mTOR Expression in Recurrence Following Hepatic Metastasectomy in Colorectal Cancer
Source: Life (Basel). 2025 May 29;15(6):877. doi: 10.3390/life15060877 (PMC12194524; doi:10.3390/life15060877)

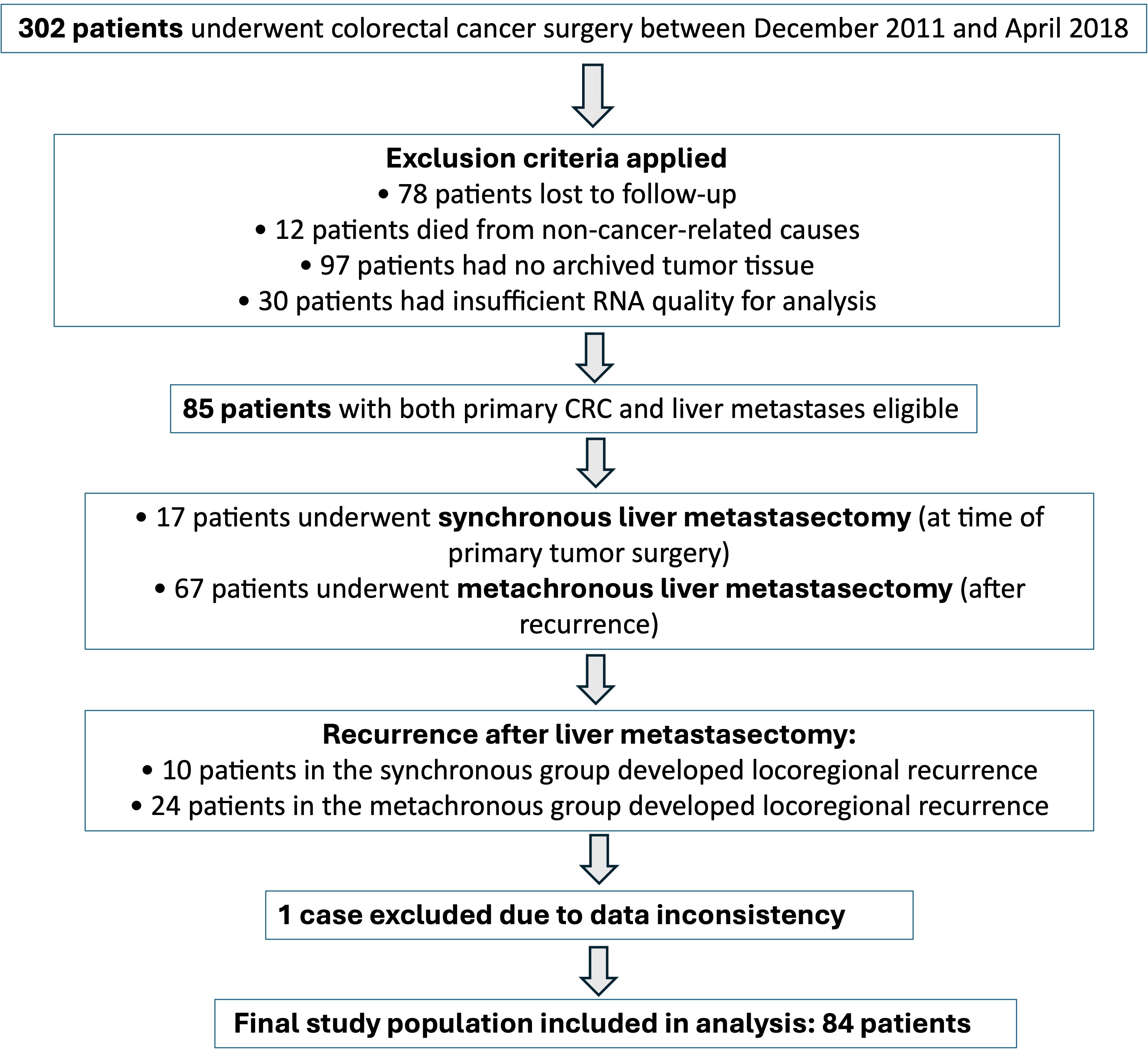


**Figure S1.** Flowchart illustrating patient selection and inclusion in the study.

Supplement: Supplementary file 1 [file life-15-00877-s001.zip › life-3631276-supplementary.docx]
